# Behavioral and Clinical Correlates of Poor Sleep and Anxiety Symptoms in Adults Aged 50 Years and Older: A Cross-Sectional Study

**DOI:** 10.3390/jcm15176531

**Published:** 2026-08-24

**Authors:** Hammad S. Alhasan, Mansour Abdullah Alshehri

**Affiliations:** Department of Medical Rehabilitation Sciences, Faculty of Applied Medical Sciences, Umm Al-Qura University, Makkah 24382, Saudi Arabia

**Keywords:** sleep quality, anxiety symptoms, generalized anxiety disorder-7, physical activity, Global Physical Activity Questionnaire, multimorbidity, musculoskeletal pain, adults aged 50 years and older, Saudi Arabia, cross-sectional study, community-dwelling adults

## Abstract

**Background/Objectives**: Sleep quality, anxiety symptoms, physical activity, musculoskeletal pain, and multimorbidity are interrelated health domains, but evidence examining these factors together among adults aged ≥50 years in Saudi Arabia remains limited. This study described their prevalence and assessed cross-sectional associations among these domains. **Methods**: A cross-sectional online survey using convenience sampling was conducted in Saudi Arabia between December 2024 and April 2025. Participants completed measures of musculoskeletal pain, self-reported morbidity, physical activity (Global Physical Activity Questionnaire), sleep quality (Brief Version of the Pittsburgh Sleep Quality Index), and anxiety symptoms (Generalized Anxiety Disorder-7). Associations were examined using Spearman rank correlations and logistic regression with core adjustment for age, sex, and BMI. The Benjamini–Hochberg false discovery rate correction was applied to eight focal comparisons; Firth penalized logistic regression was applied as a sensitivity analysis for models in which multimorbidity was the outcome. **Results**: The sample included 298 adults (mean age 58.2 +/− 6.3 years; 75.5% male). Musculoskeletal pain was reported by 73.8%, multimorbidity by 8.4%, poor sleep by 43.3%, and moderate-to-severe anxiety symptoms by 23.8%. Better sleep quality was moderately associated with lower anxiety severity (Spearman rho = −0.45, *p* < 0.001). Poor sleep was associated with higher odds of moderate-to-severe anxiety symptoms (OR 6.49, 95% CI 3.45–12.20), and low total physical activity was associated with higher odds of both moderate-to-severe anxiety symptoms (OR 7.87, 95% CI 2.68–23.08) and poor sleep (OR 5.09, 95% CI 2.40–10.83). These focal associations remained significant after FDR correction. In an exploratory sensitivity analysis, the poor sleep–multimorbidity association remained comparable when Firth regression was applied (OR 2.71, 95% CI 1.12–7.06; *p* = 0.027). **Conclusions**: In this online convenience sample of adults aged ≥50 years, sleep quality, anxiety symptoms, and total physical activity showed consistent cross-sectional associations. Findings involving multimorbidity were exploratory because of the limited number of events. These results require confirmation in representative longitudinal studies.

## 1. Introduction

The global population is ageing at an accelerating pace, placing increasing demands on health systems as chronic conditions more often co-occur within individuals rather than presenting in isolation [1,2]. Multimorbidity, commonly defined as the coexistence of ≥2 long-term conditions, is prevalent in community-dwelling populations, with prevalence increasing with age and varying markedly across populations and settings [1,3]. This clustering of conditions is clinically significant because multimorbidity is associated with greater functional impairment, increased healthcare utilization, and more complex self-management demands compared with single-disease approaches to care [4,5,6].

Similarly, musculoskeletal conditions contribute substantially to the global burden of disability. The Global Burden of Disease 2021 analysis reported low back pain as the largest contributor to global years lived with disability [7], highlighting its substantial impact on mobility, independence, and quality of life across the adult lifespan, including later life [7,8,9]. Musculoskeletal pain commonly co-exists with cardiometabolic and other chronic conditions, and may amplify disability by lowering activity tolerance, contributing to deconditioning, and constraining participation in daily and social roles [10,11,12]. Beyond the burden of disease, modifiable behavioural and psychosocial factors such as physical activity, sleep, and anxiety are increasingly acknowledged as central to healthy ageing [13,14,15]. Sleep health is equally critical: sleep disturbance is common in later life and is increasingly associated with physical and mental health outcomes such as chronic disease accumulation and emotional distress [16,17].

Growing evidence suggests these domains are interrelated rather than independent [18]. It indicates that poor sleep quality and inadequate sleep duration are consistently linked to multimorbidity across diverse settings [19]. Prospective studies have also reported longitudinal associations between sleep parameters and subsequent multimorbidity [19], as well as bidirectional relationships between sleep disturbance and anxiety symptoms [18]. These findings generate hypotheses regarding potential temporal pathways, whereas the present cross-sectional study was designed to examine concurrent associations only. Collectively, these findings support evaluating physical activity, sleep, pain, anxiety symptoms, and multimorbidity within a multidomain framework while maintaining a clear distinction between cross-sectional association and temporal interpretation.

In Saudi Arabia, population ageing is occurring alongside a high burden of non-communicable diseases in adults aged 50 years and older; national primary care records indicate a high prevalence of diabetes, hypertension, and obesity among adults attending primary care clinics [20,21]. Similarly, national screening efforts suggest that anxiety symptoms are a measurable public health concern, with incomplete detection and treatment [22,23]. Nevertheless, evidence remains scarce on how multimorbidity, recent musculoskeletal pain, physical activity, sleep quality, and anxiety symptoms cluster and are concurrently associated among adults aged ≥50 years in Saudi Arabia. Specifically, few studies have evaluated these clinical, behavioral, and psychological domains concurrently using standardized measures within a unified analytical framework. Accordingly, this study aimed to (1) characterize the prevalence of multimorbidity, recent musculoskeletal pain, physical activity levels, poor sleep, and anxiety symptoms among adults aged ≥50 years residing in Saudi Arabia; and (2) evaluate the cross-sectional associations among these clinical and behavioral measures.

## 2. Materials and Methods

### 2.1. Study Design

This study was a cross-sectional web-based survey of adults aged ≥50 years residing in Saudi Arabia, conducted using non-probability convenience sampling. The web-based questionnaire was disseminated during the data-collection period via social-media and community communication platforms. Approval for the study was granted by the Biomedical Ethics Committee of Umm Al-Qura University, Saudi Arabia (HAPO-02-K-012-2024-12-2352). All procedures followed the ethical standards outlined in the Declaration of Helsinki, and reporting was prepared in alignment with the STROBE recommendations for observational studies.

### 2.2. Participants and Eligibility Criteria

Eligible individuals were residents of Saudi Arabia aged 50 years or older. To be included, respondents needed sufficient ability to understand the questionnaire and complete the online form independently or with limited support. Adults aged <50 years were excluded. Consent was provided electronically prior to starting the survey. Participation was optional, and confidentiality safeguards were applied to preserve privacy and ensure the anonymous handling of data during collection and subsequent analyses.

### 2.3. Data Collection

Data were collected using a web-based questionnaire administered through SurveyMonkey. The survey link was distributed through social media platforms and community-based communication channels and remained open for five months (December 2024 to April 2025). Participants had access to support through a dedicated support line (telephone, WhatsApp, or email). When required, the survey could be completed by telephone with a member of the research team, following a standardized script to maintain consistency.

**Figure 1:**
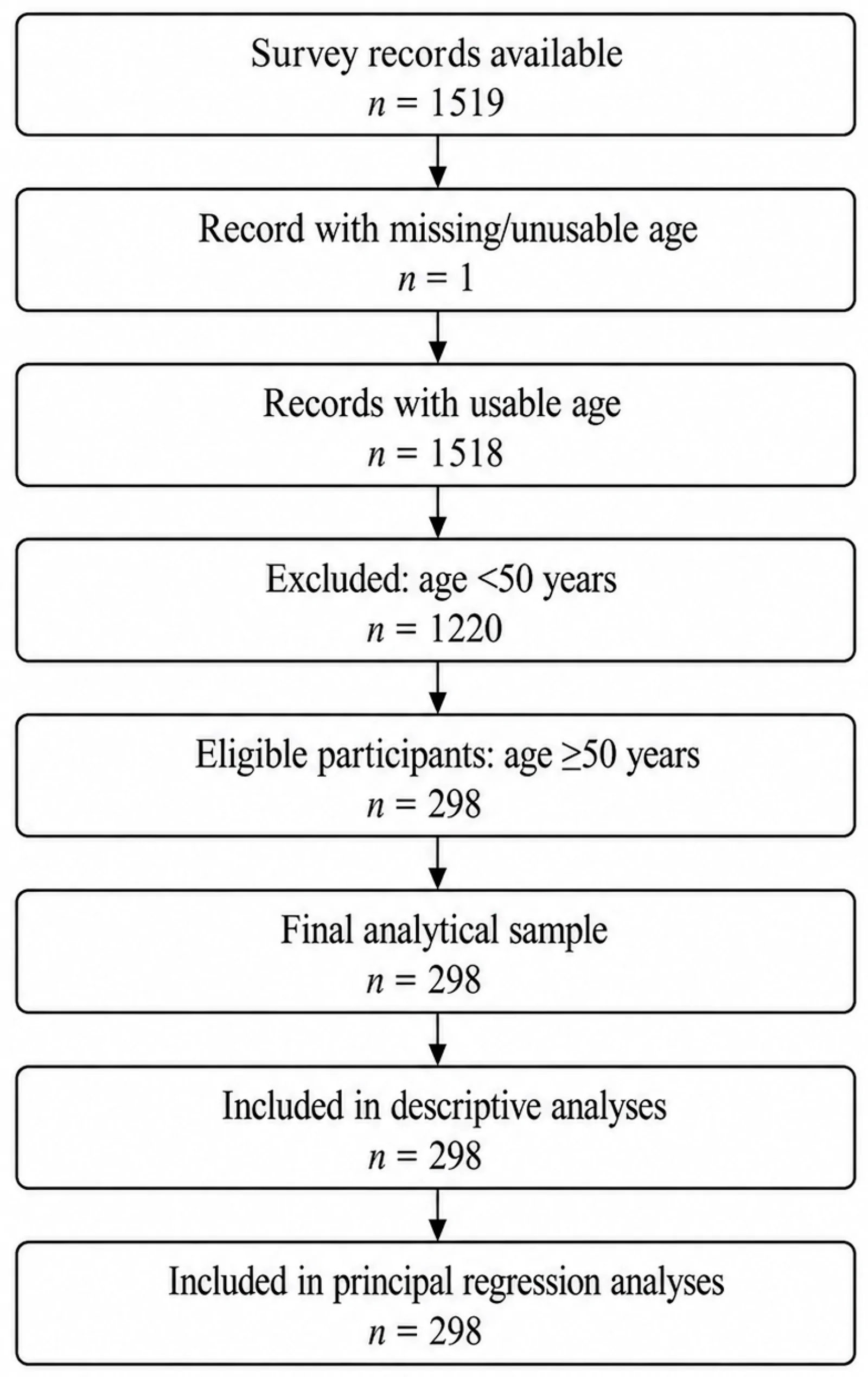
Participant flow diagram.

An online participant information sheet was presented before the questionnaire, detailing the study aims, procedures, confidentiality protections, and voluntary participation with the right to withdraw at any time; participants provided electronic informed consent before proceeding. Survey responses were retained on a secure, password-restricted system, with access limited to the study investigators. The survey dataset contained 1519 records. Information on individuals who may have accessed the survey link but did not produce an exported survey record was not retained. Participant selection and the derivation of the final analytic sample are presented in Figure 1.

### 2.4. Survey

The survey was designed to reduce response bias by presenting items in a structured sequence and covered four main domains:

#### 2.4.1. Sociodemographic and Lifestyle Variables

Participants reported age, sex, living arrangement, marital status, educational level, employment status, and work environment. Smoking status was recorded, and body mass index (BMI) was computed from self-reported weight and height.

#### 2.4.2. Musculoskeletal Pain and Morbidities

Musculoskeletal pain was recorded as the self-reported presence or absence of pain during the previous week. This measure reflected recent pain status rather than chronic pain burden and did not quantify pain severity, duration, anatomical distribution or analgesic use. Participants also self-reported health conditions from a predefined checklist (Table A1). Conditions were self-reported and were not independently confirmed as physician-diagnosed. The analytical morbidity variable contained two coded levels: a lower morbidity category and multimorbidity, defined as ≥2 conditions. A separate zero-condition level was not retained in the final analytical variable. Therefore, “lower morbidity category” is used as the reference label throughout. The musculoskeletal pain and morbidities checklist has been published previously [24].

#### 2.4.3. Physical Activity

Physical activity was measured using the GPAQ, which assesses activity performed in three domains: work, transport, and leisure-time. Additionally, an overall physical activity level was derived by combining activity across all domains. For this study, GPAQ outputs were categorized into three levels (Low/Moderate/High) for each of the three domains. These categories are interpreted as follows [25]:

Low: insufficient activity to meet recommended thresholds, reflecting a predominantly inactive pattern.

Moderate: meets basic recommended activity targets through regular moderate activity and/or some vigorous activity.

High: a high-volume activity profile, typically reflecting frequent and/or sustained vigorous activity or a large accumulated weekly activity volume.

For regression analyses, categorical-variable handling depended on the model specification. Binary low versus moderate/high coding was applied where necessary to improve model stability. Transport-related regression models remained non-estimable because of sparse cells/separation and were therefore not interpreted. The original three-level total-activity variable was also retained in sensitivity analyses to assess whether dichotomization altered the main conclusions.

#### 2.4.4. Sleep and Anxiety

Sleep quality was assessed using the six-item Brief Version of the Pittsburgh Sleep Quality Index (B-PSQI), which yields five scored components and a global score ranging from 0 to 15, with higher scores indicating poorer sleep quality [26]. The B-PSQI validation study reported good reliability and validity, including sensitivity of approximately 75.8% and specificity of 77.0% for identifying poor sleepers at a global score > 5. Accordingly, scores > 5 were classified as poor sleep and scores ≤ 5 as good sleep. This threshold was adopted from the original B-PSQI validation study and has not been specifically validated in Saudi adults aged ≥50 years [27].

Anxiety symptoms were measured using the GAD-7 scale, a brief screening instrument comprising seven items evaluating the frequency of anxiety-related symptoms over the prior two weeks. Responses are recorded on an ordinal frequency scale and summed to produce a total severity score. Participants were initially classified into four anxiety severity levels (minimal [range: 0–4], mild [range: 5–9], moderate [range: 10–14], and severe [range: 15–21]) [28]. However, because the severe GAD-7 category included only 11 participants, when GAD-7 was modelled as a binary outcome, the four-category classification was dichotomized as minimal/mild versus moderate/severe to improve estimation stability. The original four-category GAD-7 severity classification was retained in an ordinal sensitivity analysis. Accordingly, GAD-7 was recorded as a binary variable: minimal/mild (range: 0–9) versus moderate/severe (range: 10–21) anxiety. These levels were interpreted as reflecting an increasing gradient of symptom severity. For inferential purposes, the moderate/severe category was used as a screening indicator of moderate-to-severe anxiety symptoms; GAD-7 scores were not interpreted as establishing a clinical anxiety-disorder diagnosis.

### 2.5. Sample Size and Model Adequacy

Sample adequacy for the conventional logistic regression analyses was assessed using outcome-specific event counts and the events-per-variable (EPV) framework as a descriptive indicator of model information rather than as a strict adequacy threshold [29]. Each core-adjusted model included one factor of interest together with age, sex, and BMI; the number of model parameters depended on the coding of the factor of interest. Age and sex contributed one model parameter each, while BMI was specified using two indicator parameters; therefore, each adjusted model included five non-intercept parameters, including the factor of interest. The corresponding event counts and estimated EPV values were 25 multimorbidity events (EPV = 5.0), 45 moderate/high work-activity events (EPV = 9.0), 95 moderate/high leisure-activity events (EPV = 19.0), 154 low-total-activity events (EPV = 30.8), 129 poor-sleep events (EPV = 25.8), and 71 moderate-to-severe anxiety-symptom cases (EPV = 14.2). After dichotomization, the combined moderate/high transport-activity category included 73 participants; however, some transport-activity models were non-estimable because of sparse combinations of model factors and separation in the data and were therefore not interpreted further. The limited model information available for multimorbidity, and to a lesser extent work-activity models, was acknowledged through their cautious exploratory interpretation. Estimates from models with sparse outcomes were interpreted with caution, with emphasis on confidence-interval width and model stability.

### 2.6. Statistical Analysis

Primary descriptive and conventional regression analyses were performed using IBM SPSS Statistics version 27. Statistical tests were two-sided. The final analytical dataset included complete data for the variables used in the reported primary analyses; therefore, the principal estimable regression models were based on *N* = 298 and no additional within-model case deletion was required for these variables.

#### 2.6.1. Preliminary Data Handling and Variable Coding

Variables were coded for analysis using prespecified categories. Age was analysed as a continuous variable for regression models. Sex was coded as male/female. BMI was categorized as normal weight (18.5–24.9), overweight (25.0–29.9), and obese (≥30.0). Smoking status was coded as never smoked vs. current smoker. Additional sociodemographic variables were categorized to support group-based comparisons and regression modelling, including living arrangement (with family vs. alone), marital status (engaged/married vs. widowed/separated/single/divorced), employment status (working vs. retired/unemployed), and work environment (office-based vs. field-based). Primary outcomes were evaluated using the prespecified binary classifications. The modelled binary outcome contrasts were multimorbidity (≥2 conditions vs. lower morbidity category), moderate/high work activity (vs. low), moderate/high leisure-time activity (vs. low), low total activity (vs. moderate/high), poor sleep (vs. good sleep), and moderate/severe anxiety symptoms (vs. minimal/mild symptoms). Model contrasts are stated explicitly as the first category relative to the second category.

#### 2.6.2. Bivariate Comparisons and Association Testing

Spearman rank correlation coefficients were used to assess associations among age and the coded clinical and behavioral measures. Age was analyzed continuously. Musculoskeletal pain was coded as 0 = no and 1 = yes; morbidity was coded as 0 = lower morbidity category and 1 = multimorbidity; total physical activity was coded as 1 = low, 2 = moderate, and 3 = high; sleep quality was coded as 1 = poor and 2 = good; and anxiety severity was coded as 1 = minimal, 2 = mild, 3 = moderate, and 4 = severe. Thus, the direction of each Spearman coefficient reflects the stated coding direction. Specifically, a negative correlation between sleep quality and anxiety severity indicates that better sleep was associated with lower levels of anxiety symptoms.

#### 2.6.3. Logistic Regression Modelling

Binary logistic regression was used to estimate odds ratios (ORs) with 95% confidence intervals (CIs) for associations between modeled factors and binary outcomes. A uniform modelling approach was used across outcomes: unadjusted models, in which each modeled factor was entered individually to obtain crude ORs, and core-adjusted models, in which each factor of interest was entered individually together with the core covariates age, sex, and BMI; different health and behavioural factors were not included simultaneously into a single fully adjusted model. Because all variables were assessed contemporaneously, specification of a variable as an outcome in a specific model reflects the statistical model specification only and should not be interpreted as establishing temporal directionality. Age, sex, and BMI were specified as a consistent core covariate set to provide parsimonious adjustment across outcome models while limiting model complexity. The resulting estimates are therefore interpreted as core-covariate-adjusted associations rather than fully confounder-adjusted or causal effects.

Regression results were reported as odds ratios (ORs) with 95% confidence intervals (CIs) and *p*-values. Because multimorbidity was observed in only 25 participants, models in which multimorbidity was the outcome were also evaluated using Firth penalized logistic regression, while retaining adjustment for age, sex, and BMI. These analyses were considered exploratory.

To evaluate the effect of dichotomization, sensitivity analyses retained the original categorical structure where feasible. GAD-7 severity was evaluated using a four-category proportional-odds ordinal logistic regression model, and total GPAQ activity was also examined using its original low, moderate, and high categories.

Eight focal adjusted comparisons involving poor sleep and moderate-to-severe anxiety symptoms were highlighted, and their *p*-values were further adjusted using the Benjamini–Hochberg false discovery rate (FDR) procedure. Additional reciprocal and domain-specific physical-activity models were treated as exploratory.

Sex–exposure interaction terms were evaluated for the principal sleep-anxiety and total-physical-activity associations to assess potential sex-based heterogeneity. Interaction models were adjusted for age and BMI.

## 3. Results

### 3.1. Sociodemographic and Lifestyle Characteristics

Of 1519 survey records, one lacked valid age data and 1220 respondents were aged <50 years, resulting in 298 participants aged ≥50 years who were included in the analysis. Participants were aged 50–85 years (mean 58.2 ± 6.3 years). Most participants were male (75%) and either overweight (47%) or obese (32%). Musculoskeletal pain was reported by 73% of participants. For chronic conditions, 91% were classified in the lower morbidity category and 8% in the higher multimorbidity (≥2 conditions) category. The frequency of each self-reported condition included in the morbidity checklist is reported in Appendix A Table A1. In terms of physical activity (GPAQ overall activities), 51% had low activity, 30% moderate activity, and 17% high activity. Sleep quality was poor in 43% of participants and good in 56%. Anxiety severity (GAD-7) was minimal in 47.0%, mild in 29%, moderate in 20%, and severe in 3% of the sample. For detailed sociodemographic, clinical, and lifestyle characteristics of the sample, refer to Table 1. Age-group-specific descriptive characteristics for participants aged 50–59 years and ≥60 years are reported in Appendix A Table A2.

### 3.2. Correlation Analysis of Clinical and Behavioural Measures

**Figure 2:**
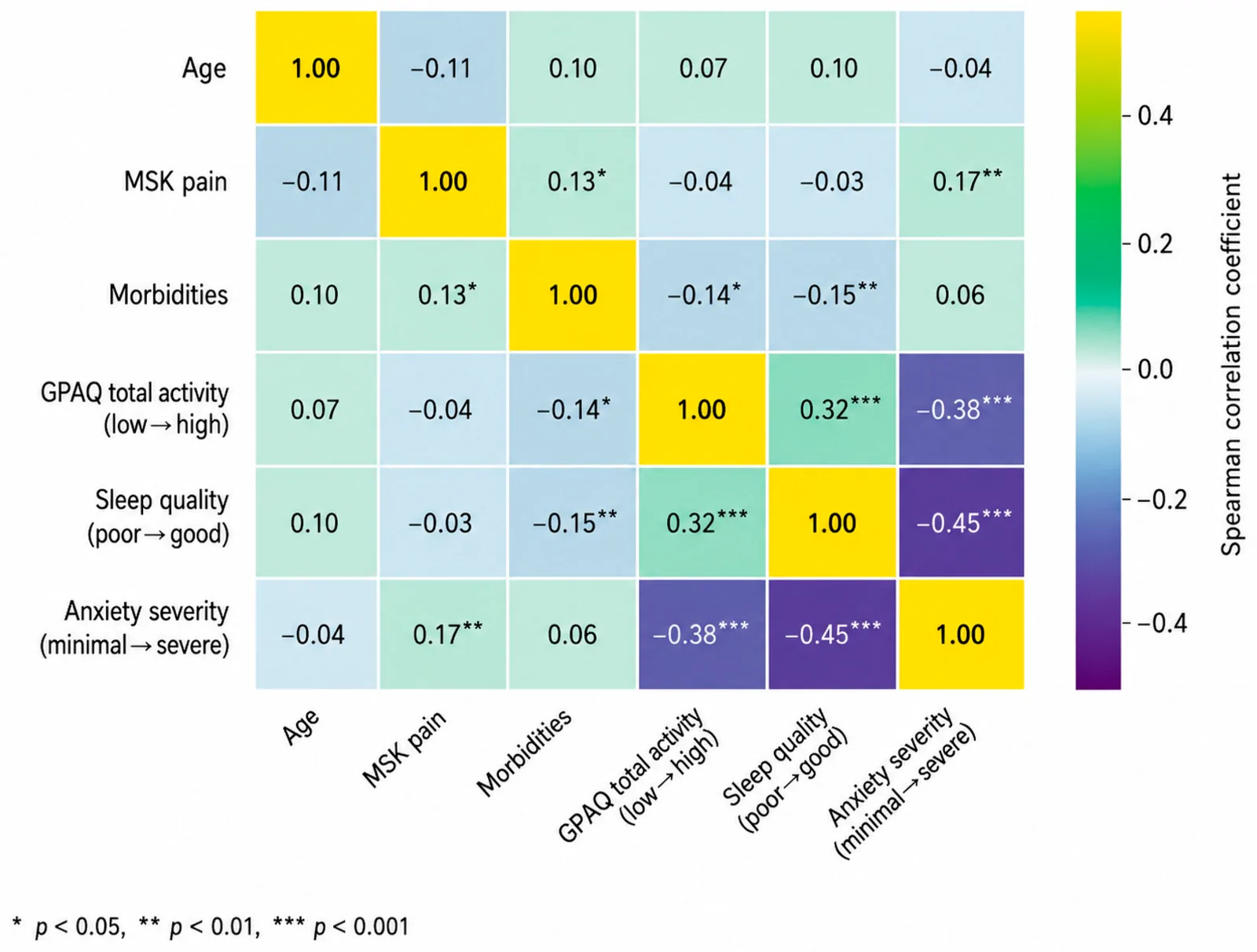
Spearman correlation matrix of the main study variables. Values represent Spearman’s rho. “GPAQ total activity (low -> high)”, “Sleep quality (poor -> good)”, and “Anxiety severity (minimal -> severe)”. “Ordered-category coding: GPAQ total activity, low to high; sleep quality, poor to good; anxiety severity, minimal to severe. Thus, negative sleep-anxiety correlations indicate that better sleep was associated with lower anxiety severity”.

Spearman’s rank correlations indicated that age had only weak and non-significant associations with multimorbidity, musculoskeletal pain, physical activity, sleep quality, and anxiety severity (all rho ≤ 0.14, *p* > 0.05). Multimorbidity showed a positive association with reported musculoskeletal pain (rho = 0.13, *p* = 0.031) and poorer sleep quality (rho = −0.15, *p* = 0.009) and showed a small negative correlation with total physical activity (rho = −0.14, *p* = 0.014). Musculoskeletal pain was not significantly related to sleep quality or total physical activity but showed a small positive correlation with anxiety (rho = 0.17, *p* = 0.003). Sleep quality and anxiety severity showed a moderate inverse correlation (rho = −0.45, *p* < 0.001). Greater total physical activity was moderately associated with better sleep quality (rho = 0.32, *p* < 0.001) and lower anxiety severity (rho = −0.38, *p* < 0.001). These relationships are summarized in Figure 2.

### 3.3. Focal and Exploratory Logistic Regression Analyses

Eight focal adjusted comparisons involving poor sleep and moderate-to-severe anxiety symptoms are shown in Table 2. Additional reciprocal and domain-specific models are reported as exploratory in Appendix A Table A3.

#### 3.3.1. Exploratory Analyses with Multimorbidity as the Outcome

In the core-adjusted logistic regression model, poor sleep was associated with increased odds of multimorbidity (OR 2.88, 95% CI 1.12–7.38; *p* = 0.028). This estimate was materially similar in the Firth penalized logistic-regression sensitivity analysis (OR 2.71, 95% CI 1.12–7.06; *p* = 0.027). The absence versus presence of musculoskeletal pain was not significantly associated with multimorbidity (OR 0.27, 95% CI 0.06–1.28; *p* = 0.099).

In the unadjusted analysis, the absence versus presence of musculoskeletal pain was associated with lower odds of multimorbidity (OR 0.23, 95% CI 0.05–0.98; *p* = 0.047). Other Firth sensitivity estimates were not statistically significant (Table 3).

#### 3.3.2. Focal Analyses with Moderate-to-Severe Anxiety Symptoms as the Outcome

Poor sleep was associated with increased odds of moderate-to-severe anxiety symptoms (OR 6.49, 95% CI 3.45–12.20; *p* < 0.001; FDR q ≤ 0.003), and low total physical activity, compared with high total physical activity, was also associated with increased odds of moderate-to-severe anxiety symptoms (OR 7.87, 95% CI 2.68–23.08; *p* < 0.001; FDR q ≤ 0.003). Multimorbidity and recent musculoskeletal pain were not statistically significant in these focal binary models. Domain-specific leisure- and work-related physical activity analyses are reported as exploratory in Appendix A Table A3.

#### 3.3.3. Focal Analyses with Poor Sleep as the Outcome

Multimorbidity was associated with increased odds of poor sleep (OR 2.87, 95% CI 1.13–7.29; *p* = 0.026; FDR q = 0.042), and low total physical activity, compared with high total physical activity, was also associated with increased odds of poor sleep (OR 5.09, 95% CI 2.40–10.83; *p* < 0.001; FDR q ≤ 0.003). Minimal anxiety symptoms were associated with reduced odds of poor sleep compared with severe anxiety symptoms (OR 0.20, 95% CI 0.05–0.75; *p* = 0.017; FDR q = 0.034). Recent musculoskeletal pain was not statistically significant in the focal poor-sleep model.

#### 3.3.4. Sensitivity and Interaction Results

Sensitivity analyses preserving the original category structure were broadly consistent with the binary analyses. In proportional-odds ordinal logistic regression, poor sleep (OR 6.08, 95% CI 3.75–9.85; *p* < 0.001) and low total physical activity (OR 4.69, 95% CI 2.94–7.47; *p* < 0.001) were associated with greater odds of higher anxiety severity. Musculoskeletal pain was also associated with greater overall anxiety severity (OR 1.95, 95% CI 1.15–3.30; *p* = 0.013).

Compared with low activity, both moderate and high activity levels were associated with reduced odds of poor sleep and moderate-to-severe anxiety symptoms (Table 4). Exploratory sex-interaction analyses provided no evidence that the sleep–anxiety or total physical activity associations differed significantly by sex (all interaction *p* ≥ 0.367; Appendix A Table A4).

## 4. Discussion

In this cross-sectional online convenience sample of adults aged ≥50 years living in Saudi Arabia, musculoskeletal pain was highly prevalent (73%), while multimorbidity was comparatively uncommon (8%). Findings from the correlation and regression analyses demonstrated consistent contemporaneous associations among sleep quality, anxiety symptoms, and physical activity, with lower physical activity levels associated with poorer sleep and greater anxiety symptom severity. These patterns are consistent with an emerging framework in ageing research that links health behaviours, sleep, and mental health.

### 4.1. Musculoskeletal Pain Findings and Interpretation

The recent-pain measure was not significantly associated with poor sleep, low total activity, or the moderate-to-severe anxiety outcome in the primary adjusted analyses. Nevertheless, the ordinal GAD-7 sensitivity analysis indicated that participants with reported musculoskeletal pain had higher overall anxiety severity. The non-significant findings from the primary binary models contrast with previous evidence linking chronic pain with poor sleep and psychological distress [29,30]. Previous research also indicates that pain-related interference and pain-related disability burden, as well as widespread rather than localized pain patterns, may be more strongly related to sleep outcomes than pain presence alone [31,32]. Additionally, the high prevalence of self-reported pain (73.8%) may have limited between-group variation. These measurement characteristics may have attenuated observable associations, and the null findings from binary models should not be interpreted as evidence that musculoskeletal pain is unrelated to sleep, activity, or anxiety symptoms.

### 4.2. Interlinked Sleep, Anxiety and Chronic Disease

The sleep–anxiety association showed the largest effect sizes, with poor sleep associated with substantially higher odds of moderate-to-severe anxiety symptoms. The present cross-sectional findings cannot establish temporal directionality. Prior longitudinal studies have documented prospective relationships between poor sleep and subsequent anxiety symptoms, with some evidence indicating that poor sleep may precede subsequent increases in anxiety symptoms more strongly than the reverse pathway [18]. Accordingly, poor sleep is discussed here as an associated clinical domain rather than as an upstream determinant or intervention target established by this study.

Multimorbidity was associated with higher odds of poor sleep (OR 2.87), and poor sleep was similarly associated with higher odds of multimorbidity. This cross-sectional association is consistent with systematic evidence showing that multiple dimensions of sleep (sleep quality, insomnia symptoms, and short/long sleep duration) are associated with multimorbidity in community-dwelling adults [33]. Furthermore, prospective cohort studies with longitudinal follow-up indicate that poor sleep may precede subsequent multimorbidity; reduced sleep duration in midlife has been associated with a greater subsequent risk of developing multimorbidity later in life [34]. Several plausible mechanisms may partly explain the observed association between multimorbidity and poor sleep. Potential contributors include increased nocturnal symptom burden (e.g., dyspnea in cardiopulmonary disease, nocturia, and discomfort), sleep fragmentation related to pain and limited mobility, and polypharmacy-related sleep disruption. Such mechanisms may impair sleep continuity and efficiency and reduce perceived sleep quality. The reciprocal sleep–multimorbidity associations are consistent with a shared and potentially reinforcing clinical relationship but cannot determine whether poor sleep precedes multimorbidity, multimorbidity contributes to sleep disturbance, or both are explained by unmeasured factors. Determining directionality and mediating pathways will require longitudinal designs and analytic frameworks (e.g., cross-lagged models) that can assess whether sleep disruption precedes subsequent disease burden, whether disease burden precedes worsening sleep, and the extent to which activity and mental health act as mediators.

### 4.3. Limitations and Future Directions

This study has several limitations. First, the cross-sectional design precludes causal inference and cannot establish temporal ordering among sleep quality, anxiety symptoms, physical activity, musculoskeletal pain, and multimorbidity. Reciprocal regression results should therefore be interpreted as contemporaneous interrelationships rather than directional effects. Second, recruitment relied on non-probability, predominantly online convenience sampling. Platform-level data on individuals who opened the survey without producing an exported record were not retained. The sample was also predominantly male (75.5%) and included a substantial proportion in the 50–59-year age group. Accordingly, the findings should not be generalized to the Saudi population aged ≥50 years as a whole, especially women, the oldest-old adults, individuals living alone, and people with limited digital engagement. The sample should also not be interpreted as representative of a typical older-adult population.

Third, key measures were self-reported. The morbidity checklist was not independently confirmed as physician-diagnosed, and the B-PSQI threshold was adopted from the original validation study rather than specifically validated in Saudi adults aged ≥50 years. Musculoskeletal pain was measured only as recent presence/absence and did not capture intensity, duration, anatomical distribution, functional interference, or analgesic use, which may have attenuated associations. Fourth, binary recoding of GAD-7 and GPAQ may have reduced information and masked dose–response patterns. Sensitivity analyses retaining the original ordinal or category structure were largely consistent with the main sleep-anxiety and physical-activity findings.

Fifth, multimorbidity was observed in only 25 participants, resulting in limited event information relative to the number of parameters in adjusted models. Analyses in which multimorbidity was specified as the outcome were therefore interpreted as exploratory. Sixth, multiple associations were examined. FDR adjustment was applied to the focal comparisons, whereas reciprocal and domain-specific models were interpreted as exploratory; borderline findings should therefore be interpreted cautiously.

Seventh, residual confounding remains an important limitation. The models employed a consistent core adjustment for age, sex, and BMI but did not adjust for several potentially relevant factors, including depressive symptoms, medication use, including sleep medications, chronic pain severity, and specific chronic disease types. The reported odds ratios should therefore be interpreted as core-adjusted associations rather than estimates of independent causal effects. The magnitude of some odds ratios should also be interpreted with caution. Because sleep quality, anxiety symptoms, physical activity, pain, and morbidity were assessed concurrently and largely using self-reported measures, the observed associations may be influenced by common-method bias and simultaneous cross-sectional measurement. Wide confidence intervals for some exploratory associations further reflect substantial statistical uncertainty; therefore, the magnitude of individual odds-ratio estimates should not be interpreted as evidence of strong causal effects.

Finally, exploratory sex-interaction analyses did not reach statistical significance, but the smaller female sample limits power and should not be interpreted as demonstrating equivalence across sexes. Sex-specific factors related to sleep and psychological symptoms in midlife may extend beyond age and BMI, including reproductive-history characteristics reported in prior research [35]; better-powered sex-specific studies are warranted.

### 4.4. Clinical Implications and Future Directions

The observed association between sleep quality and anxiety symptoms suggests that combined assessment of these domains may warrant prospective evaluation in adults aged ≥50 years. In addition, the associations involving low total physical activity further support prospective evaluation of physical-activity assessment and intervention strategies in this population [36]. However, the present cross-sectional data cannot establish that paired screening improves clinical outcomes or that assessment of one domain should automatically trigger assessment of the other [37].

Future research may also investigate digital-health approaches for identifying and communicating behavioural and psychological health-related information. Recent work has highlighted the potential of machine-learning methods for automated evaluation of health-related information in social-media environments, underscoring opportunities for incorporating digital data and intelligent analytic approaches into future public-health research [37].

## 5. Conclusions

In this cross-sectional online convenience sample of 298 adults aged ≥50 years, poor sleep, moderate-to-severe anxiety symptoms, and low total physical activity demonstrated consistent contemporaneous associations after adjustment for age, sex, and BMI. The sleep-anxiety and total physical activity associations remained significant after FDR correction. Multimorbidity was observed in a small subgroup, and analyses in which it was the outcome were exploratory. These findings should be interpreted as hypothesis-generating and should not be extrapolated to the Saudi population aged ≥50 years as a whole.

## Figures and Tables

**Table 1:** Sociodemographic, clinical, and lifestyle characteristics.

Characteristic	Category	N	%
Age (years)	Mean ± SD (range)	58.16 ± 6.32 (50–85)	—
Sex	Male	225	75.5
	Female	73	24.5
BMI category	Normal weight	61	20.5
	Overweight	141	47.3
	Obese	96	32.2
Living arrangement	With family/roommates	282	94.6
	Alone	16	5.4
Marital status	Engaged/Married	264	88.6
	Widowed/Separated/Single/Divorced	34	11.4
Educational level	High school or less	77	25.8
	Bachelor/Diploma	141	47.3
	Higher degree (Master’s/Doctoral)	80	26.8
Employment status	Working	87	29.2
	Retired/Unemployed	211	70.8
Work environment	Office-based	167	56.0
	Field-based	131	44.0
Smoking status	Never smoked	253	84.9
	Current smoker	45	15.1
Musculoskeletal pain (any)	No	78	26.2
	Yes	220	73.8
Number of morbidities	Lower morbidity category	273	91.6
	Higher morbidity/multimorbidity category	25	8.4
GPAQ—total activities	Low	154	51.7
	Moderate	91	30.5
	High	53	17.8
Sleep quality (B-PSQI)	Poor sleep	129	43.3
	Good sleep	169	56.7
Anxiety severity (GAD-7)	Minimal	140	47.0
	Mild	87	29.2
	Moderate	60	20.1
	Severe	11	3.7

Abbreviations: N, number of participants; SD, standard deviation; BMI, body mass index; GPAQ, Global Physical Activity Questionnaire; B-PSQI, Brief Version of the Pittsburgh Sleep Quality Index; GAD-7, Generalized Anxiety Disorder-7. The morbidity variable contained a lower morbidity reference category and a multimorbidity category (≥2 conditions); a separate zero-condition level was not retained in the final analytical variable.

**Table 2:** Focal crude and adjusted logistic regression analyses with Benjamini–Hochberg false discovery rate correction.

Outcome (Event vs. Reference)	Associated Factor/Contrast	Crude OR (95% CI)	Crude *p*	Adjusted OR (95% CI)	Adjusted *p*	BH-FDR q
Poor sleep vs. good sleep	Multimorbidity (≥2 conditions) vs. lower morbidity category	3.06 (1.27–7.32)	0.012	2.87 (1.13–7.29)	0.026	0.042
	Musculoskeletal pain: Yes vs. No	1.13 (0.67–1.91)	0.639	0.85 (0.49–1.48)	0.569	0.569
	Low vs. high total activity	5.37 (2.57–11.22)	<0.001	5.09 (2.40–10.83)	<0.001	≤0.003
	Minimal vs. severe anxiety symptoms	0.16 (0.04–0.57)	0.005	0.20 (0.05–0.75)	0.017	0.034
Moderate-to-severe anxiety symptoms vs. minimal/mild symptoms	Multimorbidity (≥2 conditions) vs. lower morbidity category	1.42 (0.83–2.44)	0.200	1.40 (0.56–3.50)	0.475	0.543
	Musculoskeletal pain: Yes vs. No	1.79 (0.94–3.61)	0.087	1.61 (0.81–3.22)	0.177	0.236
	Low vs. high total activity	8.26 (2.84–24.03)	<0.001	7.87 (2.68–23.08)	<0.001	≤0.003
	Poor sleep vs. good sleep	6.44 (3.49–11.86)	<0.001	6.49 (3.45–12.20)	<0.001	≤0.003

Abbreviations: OR, odds ratio; CI, confidence interval; BH-FDR, Benjamini–Hochberg false discovery rate. Outcomes are presented as the modeled event category relative to the reference category. Associated-factor contrasts are presented as the first category relative to the second category. Crude ORs are from unadjusted models; adjusted models controlled for age, sex, and BMI. BH-FDR q-values are based on the adjusted *p*-values for the eight focal comparisons. For adjusted *p*-values reported as <0.001, q ≤ 0.003 was conservatively calculated using *p* = 0.001.

**Table 3:** Firth penalized logistic regression sensitivity analyses for multimorbidity.

Associated Factor/Contrast	Firth OR	95% CI	*p*-Value
Poor sleep vs. good sleep	2.71	1.12–7.06	0.027
Musculoskeletal pain (no vs. yes)	0.33	0.06–1.18	0.090
Moderate/severe vs. minimal/mild anxiety symptoms	1.40	0.54–3.42	0.475
Low vs. moderate/high total physical activity	2.46	0.99–6.84	0.052

Note: Multimorbidity was the outcome in all models. Firth penalized logistic regression was used because multimorbidity occurred in only 25 participants. All models were adjusted for age, sex, and BMI and were considered exploratory. OR, odds ratio; CI, confidence interval.

**Table 4:** Sensitivity analyses retaining the original GAD-7 and GPAQ categories.

Analysis/Outcome	Factor/Contrast	Adjusted OR (95% CI)	*p*-Value
Proportional-odds ordinal logistic regression: four-level GAD-7 severity	Poor sleep vs. good sleep	6.08 (3.75–9.85)	<0.001
Proportional-odds ordinal logistic regression: four-level GAD-7 severity	Low vs. moderate/high total physical activity	4.69 (2.94–7.47)	<0.001
Proportional-odds ordinal logistic regression: four-level GAD-7 severity	Multimorbidity (≥2 vs. lower morbidity category)	1.43 (0.62–3.28)	0.404
Proportional-odds ordinal logistic regression: four-level GAD-7 severity	Musculoskeletal pain (yes vs. no)	1.95 (1.15–3.30)	0.013
Binary logistic regression: poor sleep	GPAQ total activity: moderate vs. low	0.33 (0.19–0.58)	<0.001
Binary logistic regression: poor sleep	GPAQ total activity: high vs. low	0.20 (0.09–0.42)	<0.001
Binary logistic regression: moderate/severe anxiety symptoms	GPAQ total activity: moderate vs. low	0.09 (0.03–0.24)	<0.001
Binary logistic regression: moderate/severe anxiety symptoms	GPAQ total activity: high vs. low	0.13 (0.04–0.37)	<0.001

Note: All sensitivity models used *N* = 298 and were adjusted for age, sex, and BMI. The proportional-odds ordinal logistic regression retained GAD-7 severity as four ordered categories (minimal, mild, moderate, severe). The binary logistic regression retained GPAQ total physical activity as low, moderate, and high, with low activity as the reference category. OR, odds ratio; CI, confidence interval; GAD-7, Generalized Anxiety Disorder-7; GPAQ, Global Physical Activity Questionnaire.

## Data Availability

The original contributions presented in this study are included in this article further inquiries can be directed to the corresponding author.

## Abbreviations

The following abbreviations are used in this manuscript:

B-PSQI	Brief Version of the Pittsburgh Sleep Quality Index
BMI	Body mass index
CI	Confidence interval
EPV	Events per variable
GAD-7	Generalized Anxiety Disorder-7
GPAQ	Global Physical Activity Questionnaire
N	Number of participants
OR	Odds ratio
PSQI	Pittsburgh Sleep Quality Index
SD	Standard deviation
SE	Standard error
SPSS	Statistical Package for the Social Sciences
STROBE	Strengthening the Reporting of Observational Studies in Epidemiology

## Appendix A. Supplementary Analyses

This appendix contains supplemental analyses supporting the main manuscript.

**Table A1:** Prevalence of self-reported conditions included in the morbidity checklist (N = 298).

Self-Reported Condition	N	%
Hypertension	88	29.5
Diabetes mellitus	84	28.2
Asthma	14	4.7
Anemia	9	3.0
Dyslipidemia	41	13.8
Visual impairment	67	22.5
Hearing impairment	12	4.0
Urinary incontinence	14	4.7
Chronic kidney disease	7	2.3
Chronic obstructive pulmonary disease	0	0.0
Coronary artery disease	9	3.0
Arthritis	18	6.0
Osteoporosis	13	4.4
Cancer	2	0.7
Neurological disease	1	0.3
Chronic musculoskeletal pain	16	5.4
History of serious fracture	1	0.3
History of major surgery	6	2.0
Other (free-text)	30	10.1

**Table A2:** Sociodemographic, clinical, and lifestyle characteristics by age group.

Variable	Category	N (%)			*p* Value
		50–59 Years (N = 183)	≥60 Years (N = 115)	Total (N = 298)	
Sex	Male	135 (73.8)	90 (78.3)	225 (75.5)	0.380
	Female	48 (26.2)	25 (21.7)	73 (24.5)	
BMI	Normal weight	35 (19.1)	26 (22.6)	61 (20.5)	0.743
	Overweight	89 (48.6)	52 (45.2)	141 (47.3)	
	Obese	59 (32.2)	37 (32.2)	96 (32.2)	
Living arrangement	With family/roommates	172 (94.0)	110 (95.7)	282 (94.6)	0.535
	Alone	11 (6.0)	5 (4.3)	16 (5.4)	
Marital status	Engaged/Married	163 (89.1)	101 (87.8)	264 (88.6)	0.742
	Widowed/Separated/Single/Divorced	20 (10.9)	14 (12.2)	34 (11.4)	
Educational level	High school or less	50 (27.3)	27 (23.5)	77 (25.8)	0.739
	Bachelor/Diploma	84 (45.9)	57 (49.6)	141 (47.3)	
	Higher (Master’s/Doctoral)	49 (26.8)	31 (27.0)	80 (26.8)	
Employment status	Working	80 (43.7)	7 (6.1)	87 (29.2)	<0.001
	Retired/Unemployed	103 (56.3)	108 (93.9)	211 (70.8)	
Work environment	Office-based	95 (51.9)	72 (62.6)	167 (56.0)	0.070
	Field-based	88 (48.1)	43 (37.4)	131 (44.0)	
Smoking	Never smoked	149 (81.4)	104 (90.4)	253 (84.9)	0.034
	Current smoker	34 (18.6)	11 (9.6)	45 (15.1)	
Musculoskeletal pain	No	42 (23.0)	36 (31.3)	78 (26.2)	0.110
	Yes	141 (77.0)	79 (68.7)	220 (73.8)	
Morbidities	Lower morbidity category	170 (92.9)	103 (89.6)	273 (91.6)	0.313
	Higher morbidity/multimorbidity category	13 (7.1)	12 (10.4)	25 (8.4)	
GPAQ—work activity	Low	154 (84.2)	99 (86.1)	253 (84.9)	0.748
	Moderate	24 (13.1)	12 (10.4)	36 (12.1)	
	High	5 (2.7)	4 (3.5)	9 (3.0)	
GPAQ—transport activity	Low	144 (78.7)	81 (70.4)	225 (75.5)	0.271
	Moderate	38 (20.8)	33 (28.7)	71 (23.8)	
	High	1 (0.5)	1 (0.9)	2 (0.7)	
GPAQ—leisure activity	Low	135 (73.8)	68 (59.1)	203 (68.1)	0.014
	Moderate	38 (20.8)	32 (27.8)	70 (23.5)	
	High	10 (5.5)	15 (13.0)	25 (8.4)	
GPAQ—total activities	Low	101 (55.2)	53 (46.1)	154 (51.7)	0.284
	Moderate	53 (29.0)	38 (33.0)	91 (30.5)	
	High	29 (15.8)	24 (20.9)	53 (17.8)	
Sleep quality (B-PSQI)	Poor sleep	84 (45.9)	45 (39.1)	129 (43.3)	0.251
	Good sleep	99 (54.1)	70 (60.9)	169 (56.7)	
Anxiety (GAD-7)	Minimal	83 (45.4)	57 (49.6)	140 (47.0)	0.745
	Mild	56 (30.6)	31 (27.0)	87 (29.2)	
	Moderate	36 (19.7)	24 (20.9)	60 (20.1)	
	Severe	8 (4.4)	3 (2.6)	11 (3.7)	

Abbreviations: N, number of participants; BMI, body mass index; GPAQ, Global Physical Activity Questionnaire; B-PSQI, Brief Version of the Pittsburgh Sleep Quality Index; GAD-7, Generalized Anxiety Disorder-7.

**Table A3:** Exploratory reciprocal and domain-specific core-adjusted logistic regression analyses.

Outcome	Associated Factor/Contrast	Adjusted OR (95% CI)	*p*-Value
Multimorbidity	Poor sleep vs. good sleep	2.88 (1.12–7.38)	0.028
Multimorbidity	Musculoskeletal pain (no vs. yes)	0.27 (0.06–1.28)	0.099
Multimorbidity	Minimal/mild vs. moderate/severe anxiety symptoms	0.72 (0.28–1.86)	0.503
Multimorbidity	Low total activity vs. moderate/high	2.70 (0.58–12.49)	0.204
GPAQ work activity (moderate/high vs. low)	Leisure activity (moderate/high vs. low)	0.26 (0.10–0.67)	0.006
GPAQ work activity (moderate/high vs. low)	Low total activity vs. moderate/high	0.23 (0.10–0.51)	<0.001
GPAQ work activity (moderate/high vs. low)	Poor sleep vs. good sleep	0.36 (0.17–0.75)	0.007
GPAQ work activity (moderate/high vs. low)	Minimal/mild vs. moderate/severe anxiety symptoms	3.14 (1.17–8.41)	0.023
GPAQ work activity (moderate/high vs. low)	Musculoskeletal pain (yes vs. no)	1.01 (0.50–2.05)	0.978
GPAQ work activity (moderate/high vs. low)	Lower morbidity category vs. multimorbidity	0.77 (0.21–2.78)	0.695
GPAQ leisure activity (moderate/high vs. low)	Low work activity vs. moderate/high	0.09 (0.02–0.46)	0.004
GPAQ leisure activity (moderate/high vs. low)	Poor sleep vs. good sleep	0.26 (0.09–0.80)	0.018
GPAQ leisure activity (moderate/high vs. low)	Musculoskeletal pain (yes vs. no)	0.78 (0.32–1.89)	0.580
GPAQ leisure activity (moderate/high vs. low)	Minimal/mild vs. moderate/severe anxiety symptoms	3.38 (0.83–13.79)	0.089
Low total activity vs. moderate/high	Multimorbidity (≥2 vs. lower morbidity category)	2.85 (1.06–7.68)	0.039
Low total activity vs. moderate/high	Poor sleep vs. good sleep	3.57 (2.17–5.88)	<0.001
Low total activity vs. moderate/high	Moderate/severe vs. minimal/mild anxiety symptoms	9.72 (4.56–20.71)	<0.001
Low total activity vs. moderate/high	Musculoskeletal pain (yes vs. no)	0.96 (0.55–1.64)	0.868
Moderate/severe anxiety symptoms	Low leisure activity vs. moderate/high	4.86 (1.02–23.29)	0.048
Moderate/severe anxiety symptoms	Low work activity vs. moderate/high	1.27 (0.73–2.20)	0.403
Poor sleep vs. good sleep	Low leisure-time activity vs. moderate/high	4.69 (1.52–14.45)	0.007

Note: These exploratory models used N = 298 and were adjusted for age, sex, and BMI. Nominal *p*-values are shown; FDR adjustment was applied only to the eight focal comparisons in the main regression table. For multimorbidity, the musculoskeletal-pain contrast is shown as no vs. yes to reflect the verified direction of the OR of 0.27. GPAQ transport models were not estimable because of sparse categories/non-convergence. OR, odds ratio; CI, confidence interval; GPAQ, Global Physical Activity Questionnaire.

**Table A4:** Exploratory sex-interaction analyses for the principal sleep, anxiety, and physical-activity associations.

Outcome	Interaction Term	Interaction Coefficient, β (SE)	Interaction *p*-Value
Moderate-to-severe anxiety symptoms	Poor sleep × sex	0.685 (0.871)	0.431
Moderate-to-severe anxiety symptoms	Low total physical activity × sex	−0.115 (0.908)	0.899
Poor sleep	Moderate/severe anxiety symptoms × sex	0.785 (0.871)	0.367
Poor sleep	Low total physical activity × sex	−0.435 (0.591)	0.461

Note: Exploratory interaction models assessed whether the principal associations differed by sex. Models were adjusted for age and BMI and included the corresponding main effects and exposure-by-sex interaction term. None of the interaction terms was statistically significant (all *p* ≥ 0.367). Because women represented 24.5% of the sample, these analyses should be interpreted cautiously because statistical power for interaction testing was limited. β, logistic-regression interaction coefficient; SE, standard error.
